# Role of macrophages in peritoneal dialysis-associated peritoneal fibrosis

**DOI:** 10.1080/0886022X.2025.2474203

**Published:** 2025-03-05

**Authors:** Chenling Chu, Ying Huang, Luxi Cao, Shuiyu Ji, Bin Zhu, Quanquan Shen

**Affiliations:** ^a^ Department of Clinical Medicine, Hangzhou Normal University, Hangzhou, Zhejiang, China; ^b^ Urology & Nephrology Center, Department of Nephrology, Zhejiang Provincial People’s Hospital (Affiliated People’s Hospital, Hangzhou Medical College), Hangzhou, Zhejiang, China; ^c^ Department of Public Health and Preventive Medicine, Hangzhou Medical College, Hangzhou, Zhejiang, China; ^d^ Department of Nephrology, Zhejiang Provincial People’s Hospital Bijie Hospital, Bijie, Guizhou, China

**Keywords:** Peritoneal dialysis, peritoneal fibrosis, macrophage, epithelial mesenchymal transition, polarization, TGF-β/ Smad signaling pathway

## Abstract

Peritoneal dialysis (PD) can be used as renal replacement therapy when chronic kidney disease (CKD) progresses to end-stage renal disease. However, peritoneal fibrosis (PF) is a major cause of PD failure. Studies have demonstrated that PD fluid contains a significantly larger numbers of macrophages compared with the healthy individuals. During PD, macrophages can secrete cytokines to keep peritoneal tissue in sustained low-grade inflammation, and participate in the regulation of fibrosis-related signaling pathways, such as NF-κB, TGF-β/Smad, IL4/STAT6, and PI3K/AKT. A series of basic pathological changes occurs in peritoneal tissues, including epithelial mesenchymal transformation, overgeneration of neovasculature, and abnormal deposition of extracellular matrix. This review focuses on the role of macrophages in promoting PF during PD, summarizes the targets of macrophage-related inhibition of fibrosis, and provides new ideas for clinical research on delaying PF, maintaining the function and integrity of peritoneum, prolonging duration of PD as a renal replacement modality, and achieving longer survival in CKD patients.

## 1. Introduction

Chronic kidney disease (CKD) is present in 10%–13% of the population and is on a growing trend amidst the global increase in diabetes and hypertension and population aging [1]. Patients with CKD suffer from renal parenchymal cell damage, increased urinary albumin excretion, and decreased glomerular filtration rate during the course of the disease [2,3], eventually progressing to end-stage renal disease (ESRD), with a range of complications including acute left heart failure, severe hyperkalemia, gastrointestinal hemorrhage, and central nervous system disorders, which require renal replacement therapy [4,5]. Peritoneal dialysis (PD) utilizes the peritoneum as a semi-permeable membrane for solute and water exchange through osmotic pressure to discharge metabolites from the body, and has become one of the main renal replacement therapies because of its convenience, simplicity, economy, small impact on hemodynamics, and better preservation of residual renal function [5–7]. Approximately 11% of ESRD patients worldwide are treated with PD as a renal replacement modality, and the global annual growth rate of PD can be as high as 8% [8]. The use of PD is growing rapidly in developing countries, with the number of people on PD in China increasing nearly 20-fold between 1999 and 2018 [9,10]. However, PD patients are prone to complications such as peritoneal inflammation, protein-energy malnutrition, volume overload, and peritoneal fibrosis (PF) [11]. Prolonged PD results in PF, a fibroproliferative disorder, in which peritoneal tissue undergoes changes such as shedding of mesothelial cells, thickening of the submesothelial stroma, an increase in vasculature, overlapping of the vascular basement membranes, and a shift of epithelial cells to mesenchymal cells, in the course of prolonged PD treatment [12]. PF can be categorized as simple peritoneal sclerosis or encapsulated peritoneal sclerosis (EPS), based on the characteristics of the lesion and patients are routinely required to discontinue PD when they are diagnosed with EPS [13]. After 1 year of PD treatment, about 2.6% of patients had ultrafiltration failure (UFF), and after 6 years of dialysis, the incidence of patients with UFF was as high as 30% [13,14]. PF is one of the main causes of UFF in patients. In the process of exploring the causes of PF caused by PD, it was found that the percentage and absolute number of macrophages increased significantly in the dialysate of continuous ambulatory peritoneal dialysis (CAPD) patients [15,16]. The expression of transforming growth factor (TGF)-β, a classical marker of fibrosis, in macrophages was significantly increased in PD fluid of CAPD patients, suggesting that macrophages are involved in the fibrotic process of long-term PD [17]. However, the options for preventing and treating PF are still being explored. This paper provides an overview of the interactions among macrophages, cytokines and other cells in the process of PF. It also explores the role of macrophages in this process and seeks potential therapeutic targets for the prevention and treatment of PD-associated PF.

## 2. Mechanisms of PF

In the course of long-term PD treatment, the occurrence of PF is the final result of multiple factors. It is well known that myofibroblasts are important cells that produce collagen fibers in the process of fibrosis [18]. Resident fibroblasts, epithelial cells undergoing mesenchymal transition, perivascular cells and bone marrow derived cells are considered to be the main sources of myofibroblasts [19]. From research in animal models of peritoneal injury, some researchers believe that epithelial-mesenchymal transition (EMT) is the source of myofibroblasts [20,21]. Chen et al. [22] used lineage tracing to show that during PF after peritoneal injury, submesothelial fibroblasts are the precursors of myofibroblasts and that surviving mesothelial cells are the primary cells responsible for the *de novo* endothelialization response. Stimulated by chronic inflammation, myofibroblasts, regulated by macrophages, exhibit proliferative, and invasive properties and initiate reparative healing during injury repair [23,24]. Both macrophages and myofibroblasts secrete matrix metalloproteinases (MMPs) to degrade the extracellular matrix (ECM), renew collagen and remodel the interstitium [25,26]. Platelet-derived growth factor (PDGF), TGF-β, interleukin (IL)-1, interferon-γ (IFN-γ) and tumor necrosis factor-α (TNF-α), as the major cytokines promoting tissue necrosis play important roles in the fibrotic process [27].

**Figure 1.**
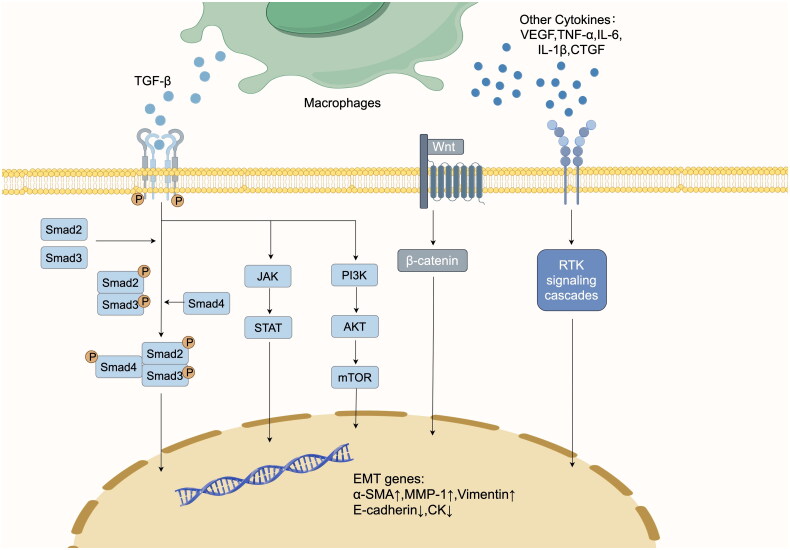
Signaling pathways in fibrotic processes involving macrophages. In the typical TGF-β/smad signaling pathway, upon release of TGF-β binds to TGFR2, which recruits and activates TGFR1. Active TGFR1 phosphorylates Smad2 and Smad3, complexes with Smad4 and translocates to the nucleus. The Smad3 component of the complex binds directly to gene promoters and induces molecules such as α-SMA. In addition, Smad3 affects miRNAs and lncRNAs, influencing epigenetic modifications of DNA and histones to encode α-SMA, vimentin and E-cadherin. In the non-Smad signaling pathways, activation of the PI3K–AKT–mTOR and JAK–STAT pathways, can affect pro-fibrotic factor gene coding. The wnt/β-catenin signaling pathway can contribute to the accumulation of β-catenin in the cytoplasm leading to the induction of MMT by nuclear translocation. Wnt3a-treated macrophages promote polarization toward M2 macrophages *via* IL-4 or TGF-β, which facilitates the development of fibrosis. Cytokines such as VEGF and CTGF bind to the receptors and regulate fibrosis.

TGF-β-enhanced expression in macrophages in PD is considered to be the main mediator of PF [28]. TGF-β was found to preferentially stimulate the synthesis of fibronectin and type I procollagen chains in fibrosis and to increase the stability of messenger RNAs encoding these 2 proteins [29]. Helmke et al. [30] found that TGF-β upregulated expression of CX3CL1 mRNA, and CX3CL1 interacted with CX3CR1 on the surface of macrophages, contributing to the expression of TGF-β, ECM, collagen and fibronectin, and advancing the development of PF. The latent associated peptide (LAP) formed by cleavage of TGF-β precursor protein is cross-linked with mature TGF-β homodimer and latent TGF-β binding protein (LTBP) to form a large complex which is stored in the ECM and forms active TGF-β after the action of protease [31]. Activated TGF-β is released and binds to TGF-β receptor (TGFR) 2, recruiting and activating TGFR1. Active TGFR1 phosphorylates Smad2 and Smad3, binds to Smad4 to form complexes and translocates to the nucleus. The Smad3 component of the complex directly binds to gene promoters and induces the expression of fibrosis markers such as α- smooth muscle actin (α-SMA). In non-Smad signaling pathways, activation of PI3K-AKT-mTOR and JAK-STAT pathways can affect the profibrotic factor gene coding [32,33] (Figure 1). Activated TGF-β activates Smad and non-Smad pathways, resulting in the transformation of peritoneal mesothelial cells (PMCs) to mesenchymal stromal cells, triggering fibrosis and immunosuppression of peritoneal tissues, and the abnormal deposition of ECM [34,35].

It was found that connective tissue growth factor (CTGF) plays an important role in the process of TGF-β-induced fibrosis [36]. CTGF, as a pro-fibrotic stromal cell protein, promotes the chemotaxis and proliferation of fibroblasts, and facilitates synthesis of the ECM, migration of endothelial cells and generation of blood vessels and lymphatic vessels [37,38]. Heat shock protein 47 (HSP47), procollagen lysine 2-oxoglutamate 5-deoxygenase (PLOD2/LH2) and prolyl-4-hydroxylase (P4HA3) can also be strongly induced by TGF-β. HSP47 is expressed in almost all cells expressing collagen I, binds to collagen and promotes structural stability in collagen assembly. Unfolded collagen strands can be further formed into stable collagen cross-links by the action of 2 key enzymes, PLOD2/LH2 and P4HA3 [39]. Peng et al. [40] used mice that overexpressed peroxisome proliferators-activated receptor γ coactivator α (mPGC-1α) in skeletal muscle as the model. Kyoto Encyclopedia of Genes and Genomes (KEGG) enrichment pathway analysis showed that TGF-β, TNF, mitogen-activated protein kinase (MAPK), Hippo, and other signaling pathways were restricted in the model of mPGC-1α inhibition of renal fibrosis, and the restriction of TGF-β signaling was significant. The important role of TGF-β signaling pathway in the process of tissue fibrosis was confirmed.

In numerous studies, TGF-β is associated with downstream signaling pathways and is involved in the induction and activation of a variety of cytokines during fibrosis in organs and tissues throughout the body, causing material deposition and structural changes. In addition, other factors can promote TGF-β expression during fibrosis, among which Smad1 and Smad3 can enhance TGF-β transcription [41]. Angiotensin II can enhance the expression of TGF-β to regulate Smads proteins and promote ECM synthesis and tissue fibrosis [26]. As can be seen from the above, TGF-β plays an important role in fibrosis. The role of TGF-β is continuously amplified by the interactions of IFN-γ, TNF-α and other cytokines, and the histological alterations associated with PF continue to accumulate, ultimately causing structural and functional alterations of the peritoneum, ultrafiltration dysfunction and gradual development of UFF.

During PD-related PF, vascular endothelial growth factor (VEGF)-A is considered to be an important cytokine mediating neovascularization during the induction of fibrosis and is regulated by TGF-β [42]. VEGF expression is elevated in peritoneal dialysate from patients with ultrafiltration problems [43]. The expression of VEGF-A mRNA in the peritoneal tissue of UFF patients was significantly increased, and VEGF-A was correlated with the number of blood vessels and the thickness of the mesodermal compact zone in the peritoneal tissue [44]. At present, researchers have used drugs to inhibit the expression of VEGF to achieve anti-fibrosis in animal models of PF [45].

Persistent low-grade inflammation is an important cause of structural changes in the peritoneum, which can lead to fibrin deposition, vascular proliferation, and fibrous capsule formation [46,47]. The inflammatory cytokines ILs, TNF-α and C-reactive protein play an important role in the process of inflammatory injury of peritoneal tissue. The expression of IL-6 and IL-1β in the dialysate of PD patients is increased [48]. IL-6 can activate the Janus kinase/signal transducer and activator of transcription (JAK/STAT) pathway to promote EMT [49]. IL-6 promotes VEGF and angiopoietin-2 through the IL-6/STAT3 signaling pathway in the PF model, thereby increasing vascular permeability and angiogenesis [49,50]. In mouse and cell models of fibrosis induced by methyl ethylene glycol and high glucose peritoneal dialysate, the inflammasome NLRP3 can regulate the release of IL-1β, mediate vascular endothelial injury and macrophage infiltration, and promote PF [51,52].

Macrophage infiltration plays an important role in the pathogenesis of PF. During chronic inflammation, molecules such as oxygen free radicals and cathepsin can be released by macrophages, causing tissue damage [53,54]. In this manuscript, the role of PD-related PF in macrophages was further explored.

## 3. The role of macrophages in PF

**Figure 2.**
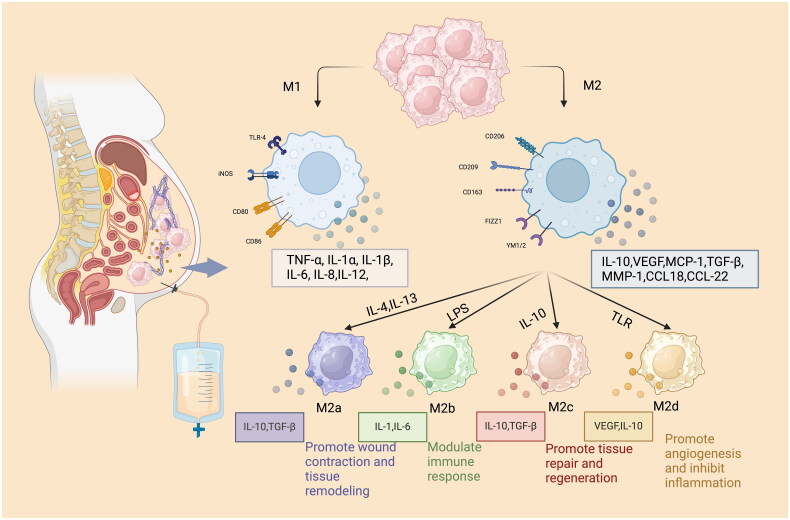
Macrophage polarization. Macrophages polarized into M1 macrophages induced by LPS, TNF-α and IL-6. M1 macrophages express TLR-4, CD80, CD86, and iNOS on the cell surface, and secrete cytokines, such as TNF-α, IL-1, IL-6, IL-8, and IL-12, to participate in tissue inflammatory response and damage PMCs. Macrophages polarized into M2 macrophages induced by IL-4 and IL-10. Cell surface expression of FIZZ1, CD163 and CD206 increased, along with secretion of cytokines such as IL-10, VEGF involved in neoangiogenesis, and pro-fibrotic cytokines such as MCP-1, CCL18 and TGF-β. M2 macrophages can be further classified and secrete different cytokines to exert biological functions.

Mononuclear phagocytes in the peritoneal immune system are mainly composed of macrophages and dendritic cells (DCs). Macrophages, as one of the major cell types in PD fluid, have a high degree of plasticity, can polarize in different microenvironment, and are involved in pathophysiological processes, such as inflammation, neoplasia, tissue repair, and metabolism [15]. Macrophages can be divided into M1 macrophages with antigen-presenting function, promoting inflammation, eliminating pathogenic microorganisms and anti-tumor function, and M2 macrophages with inhibiting inflammation, promoting tissue reconstruction and participating in angiogenesis [55]. The phenotypes of macrophages can change under different physiological environments [56,57] (Figure 2). Bellón et al. [58] used flow cytometry to examine the phenotype of macrophages in PD fluid from CAPD patients and found CD163+ and CD206+ M2 macrophages, which stimulate fibroblasts and lead to PF. On further exploration, we found that different macrophage phenotypes play different roles in PD-associated PF.

### 3.1. Mechanism of M1 macrophages during PD-related PF

**Figure 3.**
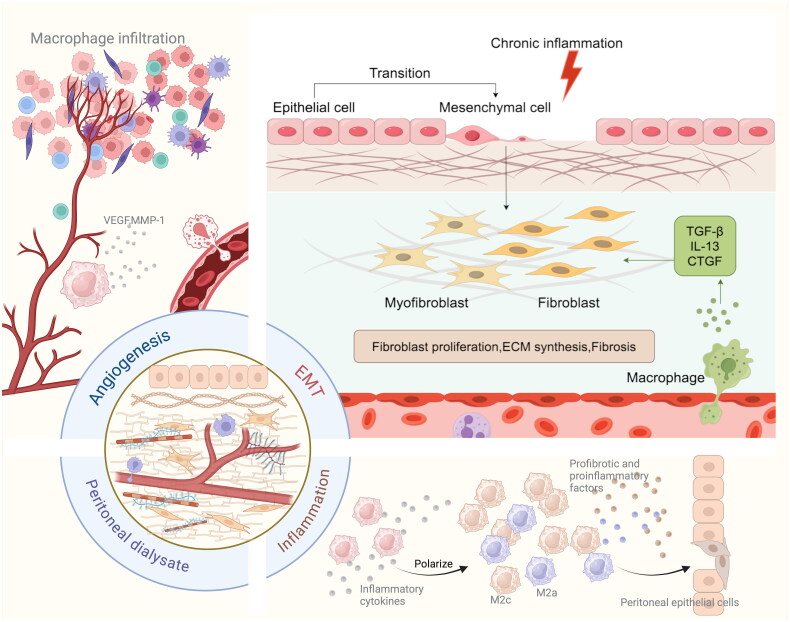
Pathophysiological process of EMT, angiogenesis, and chronic peritoneal inflammation. PMCs consist of simple squamous epithelium. Under the stimulation of chronic inflammation, epithelial cells lose apical basolateral polarity, and transform into MSCs. MSCs are capable of self-renewal and transform into fibroblasts with characteristics of migration, invasion and fibrosis. Macrophages secrete cytokines such as TGF-β, which promotes the production of collagen fibers by fibroblasts and deposition of ECM. TGF-β induces the activation of quiescent fibroblasts into myofibroblasts, which produce fibronectin and promote peritoneal fibrosis. Macrophages produce VEGF, MMP-1 and other factors to induce angiogenesis.

Habib et al. found increased expression of M1 macrophages in peritoneal tissues of EPS patients [59]. Early in the injury, macrophages are transformed into M1 macrophages by lipopolysaccharide (LPS), IFN-γ and IL-6 [60]. The effect of M1 macrophages depends on the production of IL-12 and IL-8 and expression of CD80 or CD86 on the cell surface. Guided by macrophages, inflammatory cytokines secreted by T helper (Th)1 cells can further participate in the regulation of cellular reaction of M1 macrophages [61,62]. At the early stage of peritoneal injury, macrophages activate inflammatory pathways and release inflammatory cytokines, thus keeping the peritoneum in an inflammatory state. When polarized M1 macrophages are directly co-cultured with PMCs, the PMCs lose their pebble-like morphology. Meanwhile, the mRNA expression of the mesenchymal marker α-SMA is significantly increased, while the mRNA expression of the epithelial marker E-cadherin is decreased. This indicates that M1 macrophages can promote EMT of PMCs, during which the apex of epithelial cells loses basolateral polarity. This is accompanied by a decrease in cytoskeletal proteins including cytokeratin and vimentin [38,63]. Under the stimulation of chronic inflammation, epithelial cells lose their apical basolateral polarity and transform into mesenchymal stem cells (MSCs). MSCs can self-renew and transform into fibroblasts with migration, invasion and fibrotic characteristics. Macrophages secrete TGF-β and other cytokines that promote fibroblast proliferation and ECM deposition [64] (Figure 3). The extracellular vesicles (EVs) secreted by damaged PMCs and macrophages can transmit high levels of integrin-linked kinase (ILK) to fibroblasts and induce the activation of static fibroblasts into myofibroblasts by regulating the p38 MAPK signaling pathway [65,66]. Myofibroblasts produce fibronectin and collagen fibers, and promote PF [20]. The number of EVs containing ILK is positively correlated with the degree of peritoneal injury and the duration of PD [20,66]. As a classical signaling pathway, P38 MAPK can promote macrophages to secrete inflammatory cytokines such as IL-1β and IL-6, regulate fibrotic factors such as TGF-β and FN1, ultimately induce peritoneal inflammation and peritoneal thickening in a variety of PD-induced PF models [67,68].

The inflammatory effect of M1 macrophages induces EMT in PMCs, and EMT is one of the characteristics of PF. With the further action of fibroblasts and myofibroblasts, peritoneal fibrous tissue proliferates.

The EMT process of PF depends on the direct contact between PMCs and M1 macrophages, and expression of Toll-like receptor 4 (TLR4) on the surface of M1 macrophages is increased in direct contact co-culture, indicating that TLR4 is involved in EMT as an important connection point [69]. In other studies, increased expression of TLR4 was detectable in intestinal tissues of mice that had developed cystic fibrosis and TLR4 also mediated the survival of bacteria in the tissues [70]. TLR2 and TLR4 are expressed on the surface of macrophages, and can induce tissue inflammation and activate the TGF-β1 pathway, resulting in aggravated peritoneal injury and decreased peritoneal filtration function [71,72]. TLR2 plays a significant role in PF, and its deficiency can lead to termination of fibrosis [73]. M1 macrophages also produce large amounts of proinflammatory cytokines to damage PMCS and release TGF-β1 to promote fibrosis [74,75]. M1 macrophages can induce long-term inflammation of the peritoneum through multiple pathways and molecular mechanisms in the early stage of PD, damaging the peritoneal tissue. However, under the long-term infiltration of inflammatory cells, the structural composition of PMCs changes, further decreasing the peritoneal function.

### 3.2. Mechanism of M2 macrophages during PD-related PF

The surface of M2 macrophages express CD163. The expression of CD163+ macrophages is increased in the peritoneal tissues of patients with EPS, and they infiltrate the peritoneal tissues together with CD4 + T cells to participate in EPS [59]. The expression of M2 in the peritoneal tissue of the PF mouse model was significantly increased, and the expression level of TGF-β in M2 was increased. The researchers hypothesized that M2 may promote PF by activating the TGF-β/Smad signaling pathway [76]. In clinical studies, researchers have found that M2 polarization of peritoneal macrophages in the process of PD-related PF is dependent on glucose concentration in peritoneal dialysate. During this process, arginase production may contribute to the occurrence of PF [77]. In exploring the mechanism of M2 in PD-related PF, macrophages are polarized into M2 macrophages under the action of IL-4, IL-13 and other molecules [57]. M2 macrophage surface expresses CD163 and CD206. CD206 is mainly responsible for anti-inflammatory activity and promoting fibroblast growth and collagen production. The high basal expression of CD163 on the macrophage surface is mainly involved in immunosuppression and tissue repair [78–80]. M2 macrophages secrete vascular endothelial growth factor (VEGF), IL-10, amphiregulin, and MMP-1, which can promote neovascularization, deplete CD8+ cells and reduce infiltration of inflammatory cells [81,82] (Figure 3). In sodium hypochlorite-stimulated cells and mouse models, Chen et al. found that the process of fibrosis was accompanied by M2 polarization of macrophages and increased CCL17 expression. CCL17 binds to CCR4 on submesothelial fibroblasts and stimulates fibroblast migration and collagen production [83]. In the process of PF, M2 macrophages can provide direct signals to fibroblasts for activation and multiplication, and fibroblasts continue to provide colony-stimulating factor 1, which can be used as an essential factor for the production of M2 macrophages, and intercellular mutual reinforcement, correction and promotion of complete activation [84,85] Under the regulation of protein kinase C (PKC), fibroblasts can secrete TGF-β, participate in the synthesis and secretion of ECM, collagen fibers and a large number of proteins, produce structural framework tissues, and interact positively with M2 macrophages to promote fibrosis, leading to fibrin deposition, vascular proliferation and PF. Therefore, PF does not depend on macrophages alone, and the interaction between cells is essential in this process. PD fluid drained from EPS patients Drainage fluid of EPS patients suffering from UFF during long-term PD shows increased concentration of monocyte chemotactic protein-1 (MCP-1) and more obvious expression of CCL18. IL-4 and IL-13 are secreted by Th2 cells and induce M2 macrophages to produce CCL18, which stimulates fibroblast proliferation, participates in antigen presentation, induces chemotaxis of Th2 cells, inhibits Th1 cells and plays an anti-inflammatory role [84,86,87]. In studies of human primary lung fibroblasts, sequence-specific DNA-binding protein (Sp1) was activated after CCL18 stimulation, stimulating collagen formation independent of TGF-β [88–90]. It was found that CCL18 was positively correlated with dialysate-to-plasma ratio of creatinine (D/P), and creatinine D/P reflected peritoneal permeability [58]. Therefore, CCL18, as a factor affecting PF, can be used as a marker to judge the prognosis of PD patients. The increased concentration of MCP-1, as a chemokine secreted by mononuclear macrophages, can increase the level of oxidative stress, activate NF-κB, cause inflammation-mediated tissue damage, and participate in vascular endothelial renewal and angiogenesis. MCP-1 promotes the secretion of collagen, deposition of ECM and changes of tissue structure mediated by TGF-β in PD-related PF [91,92] In conclusion, the infiltration of M2 macrophages can produce growth factors that repair the damage and play an anti-inflammatory role in promoting repair. In the late stage of peritoneal injury, M2 macrophages interact with fibroblasts and promote the occurrence of PF through PKC and TGF-β pathway with the participation of cytokines such as CCL18 and MCP-1. Therefore, the aggregation of M2 macrophages, the function of fibroblasts and the interaction of related cytokines are important processes of PF.

M2 macrophages can be further divided into M2a, M2b, M2c, and M2d. They have different surface markers and biological functions, and secrete different cytokines [93] (Figure 2). Macrophages can be induced to M2a by IL-4 and to M2c by IL-10. Both M2a and M2c can increase the expression of α-SMA in PMCs, and M2c has a more obvious role in promoting fibrosis by promoting EMT [75]. At present, the role of other subtypes of M2 macrophages in PF remains to be further studied.

## 4. Macrophages participate in cellular pathways associated with PF

In single-cell RNA sequencing results of effluent from PD patients, strong receptor-ligand correlation between various cell populations was observed. Through the intercellular communication network, ligands on fibroblasts could interact with receptors on macrophages and participate in the activation of classical pathways, including NOD-like receptor, TLR and NF-κB pathways [15]. The Wnt/β-catenin signaling pathway promotes the expression of Wnt1, Wnt5a and the accumulation of β-catenin in the cytoplasm of mesothelial cells during long-term PD, leading to nuclear translocation and inducing EMT [94–96]. This signaling pathway associated with VEGF promotes neovascularization and perpetuates low peritoneal inflammation [97]. As a soluble glycoprotein signaling molecule, Wnt3a is widely used to stimulate the Wnt/β-catenin signaling pathway. Wnt3a induces polarization into M2 macrophages through the Wnt/β-catenin signaling pathway under co-treatment of IL-4 or TGF-β and promotes fibrosis [98]. As a complex pathophysiological process, fibrosis is related to many types of cells and regulated by many factors. When the PF rat model was constructed with high glucose, PI3K, AKT and mTOR proteins were highly expressed during EMT. In the pathway, phosphorylated AKT, which is the core of the pathway, translocates to the cell membrane and activates mTOR [99]. Downstream, it promoted the polarization of M2 macrophages and regulated autophagy process through ubiquitin-proteasome 19. In the current study, autophagy is thought to be involved in PF in rats by regulating the TGF-β/Smad3 signaling pathway [100,101]. AKT can affect the TGF-β/Smad pathway by directly influencing the activity of Smad proteins in response to TGF-β [41]. When the PI3K/AKT interaction is blocked by wortmannin, expression of p-AKT and α-SMA in PMCs is significantly reduced, which inhibits PF, suggesting that the PI3K/AKT/mTOR signaling pathway is also involved in the occurrence of PF [101,102].

In the study of fibrosis, the highly conserved Hippo signaling pathway in the evolutionary process regulated the fibrosis of several organs, and the 2 main effector factors in the pathway, transcriptional regulator Yes associated protein (YAP) and co-activator PDZ-binding motif (TAZ/WWTR1), were over-activated in macrophages and promoted fibrosis. YAP activation and fibrosis-related cytokine expression are closely related to the PI3K-AKT pathway [103]. During long-term PD treatment, YAP can regulate the expression of caveolin 1 and TGF-β signaling pathway, and promote the transformation of PMCs into mesenchymal cells [104]. In cancer studies, the C-type lectin domain family 4 member E (Clec4e, also known as Mincle) of macrophages was involved in inflammation-related diseases and was strongly associated with fibrosis in adipose tissue, lung, liver and other tissues [105–107]. When activated, Mincle, as a pattern recognition receptor for damage-associated molecular patterns (DAMPs), can up-regulate the expression of collagen type I α I (COL1A1) and α-SMA genes, promote the production of collagen I and α-SMA, and interact with Fc receptors, Syk and NF-κB to promote inflammation and fibrosis [108,109]. Recombinant peroxiredoxin 1 (rPrdx1) can up-regulate the Mincle/Syk/NF-kB signaling pathway, induce and maintain the polarization of primary peritoneal macrophages to M1 macrophages, and promote expression of inflammatory cytokines [110,111].

Interactions among macrophages, fibroblasts and PMCs promote PF through the binding of ligand and receptors of various cytokines. Several cytokines associated with fibrosis can activate multiple signaling pathways and regulate macrophage polarization, providing reference targets for subsequent clinical studies of drugs to inhibit fibrosis.

## 5. PF therapeutics targeting macrophages

Due to the progress of PD technology after the 1980s, the incidence of peritonitis has declined. PF was the main reason for long-term failure of PF technology, and fibrosis was closely related to macrophages [112]. The method of reducing PF associated with PD by macrophages includes reducing M1 macrophage polarization to reduce inflammatory infiltration and inhibiting M2 macrophage polarization to reduce formation of pro-fibrotic factors.

Some research groups have found that histone deacetylase (HDAC) is directly related to STAT transcription factors, and STAT6 is a major factor affecting the polarization of M2 macrophages [113,114]. Zhou et al. [115] found that HDAC8 can inhibit cortical actin acetylation, which is involved in the regulation of actin cytoskeleton ECM degradation. PCI-34051, a specific inhibitor of HDAC8, can inhibit M2 macrophage polarization through STAT6 and the PI3K/Akt/mTOR signaling pathway, thereby inhibiting EMT and cell apoptosis and slowing PF. Another HDAC inhibitor, valproate (VPA), can reduce the polarization of M1 macrophages, reduce the production of inflammatory factors, reduce the expression surface markers CD40, CD80 of M1 macrophages and up-regulate the expression of the molecule CD86 on the surface of M2 macrophages [116]. VPA has been proven to improve postoperative peritoneal adhesion and renal fibrosis in diabetic nephropathy, and may have renal protective effects [117,118]. However, there is no direct study to prove whether VPA has an inhibitory effect in PD-related PF. LY294002 and rapamycin, as inhibitors of PI3K and mTOR, attenuated the expression of fibroblast-specific protein 1 (FSP1) and α-SMA in rat PMCs [101]. The PI3K/Akt/mTOR signaling pathway inhibits intracellular oxidative stress, promotes the expression of autophagy-related proteins LC3II/I, p62 and beclin-1, inhibits polarization to M2 macrophages and prevents fibrosis [99,101]. Ertilav et al. [119] confirmed in the study that octreotide inhibited the production and activity of TGF-β1, VEGF and MCP-1, and in a controlled trial, octreotide weakened the inflammation caused by macrophages, reduced neoangiogeneis, inhibited PF and protected peritoneal function. The differentially expressed exosome miR-204-5p, screened from the supernatant obtained from macrophage culture, was found to block the proliferation of fibroblasts, inhibit PMC EMT, and delay the PF process [120,121]. Parthenolide (PTL), as an inhibitor of NF-κB, is believed to inhibit the inflammatory response of macrophages [122]. Zhang et al. [123] demonstrated that PTL can reduce the expression of TGF-β, inhibit the phosphorylation of Smad2/3 and inhibit inflammation through the NF-κB/TGF-β/Smad signaling pathway. During this process, inflammatory factors such as IL-6, TNF-α, and MCP-1 are reduced, and EMT is inhibited, thus delaying the PF process in PD models. The progranulin currently under study can not only bind to TNF receptors to inhibit the inflammatory signaling pathway involved in TNF, but also induce the M2 phenotype during macrophage polarization, reduce the expression of TNF-α and iNOS mRNA, inhibit the NF-κB/MAPK signaling pathway and reverse the proinflammatory state [124]. Liao et al. [125] confirmed in the study that Krüppel-like factor 4 (KLF4) is a key factor regulating the polarization of macrophages. Under the action of IL-4, it collaborates with STAT6 to induce polarization of M2 macrophages and inhibits the M1 phenotype. KLF4 plays an important role in the ability of M2 macrophages to inhibit inflammation, but its mechanism in fibrosis remains controversial and is currently a potential target for the treatment of fibrosis [126]. At present, the inhibitory effect of some drugs on PF is relatively clear, but most drugs have been studied only in animal models. Due to lack of clinical trials, the efficacy and side effects of drugs still need to be further explored. Some drugs have been shown to delay fibrosis in several organs, but there is little information on the inhibition of PF.

In the study of inhibiting PF, it was found that traditional Chinese medicine and related compounds played an important role in the prevention and treatment of PF. Astragaloside and Biejijian pills regulate PF by blocking the PI3k/AKT/mTOR pathway [127,128]. Lu et al. found that quercetin inhibited the inflammatory infiltration of macrophages and the polarization of M1 macrophages in mice model of kidney injury, and the expression of iNOS, IL-12, and MCP-1 was significantly decreased after quercetin application, and the effect of kidney injury was alleviated [129]. Quercetin, as an antioxidant, can clear reactive oxygen species (ROS), which are closely related to the polarization of M1 macrophages are reduce the inflammatory response of tissues [130]. Isoliquiritigenin (ISL), a flavonoid derived from licorice, reduces macrophage secretion of inflammatory factors such as IL-1β and IL-6, as well as reducing the number of M1 macrophages [131]. ISL also inhibits the expression of Mincle genes and proteins in macrophages, and inhibits the phosphorylation of Syk and NF-κB, reducing fibrosis through the Mincle/Syk/NF-kB signaling pathway [132]. Luteolin, another flavonoid compound, has strong antioxidant and anti-inflammatory effects [133,134]. In the experimental results, luteolin can inhibit the induction of M1 macrophages by down-regulating p-STAT3, reduce the vitality of M1 macrophages, expression of CD86 on the surface of M1 macrophages and secretion of inflammatory cytokines, and increase apoptosis. However, it had no significant effect on the activity of M2 macrophages. Luteolin can induce the M2 phenotype in macrophages by up-regulating p-STAT6 and promoting the expression of IL-10 and other anti-inflammatory factors [135,136]. These drugs are shown in Table 1. Researchers have found that its glycoside analog, luteoloside, alleviates liver fibrosis through TLR2/TLR4 signaling interactions [137]. Traditional Chinese medicine and related compounds can inhibit fibrosis and inflammation, but further clinical trials are needed to clarify their effects on the peritoneum.

**Table 1.** PF therapeutics targeting macrophages.

Drug name	Animal(cell) model	Target (cell type) of action	Mechanism of action	Therapeutic effect	Refs
PF therapeutics targeting macrophages					
PCI-34051	Mice/ RAW264.7 cells	Macrophages and PMCs	Block the EGFR/ERK1/2/STAT3/HIF-1a signaling pathway in PMCs Inhibit of STAT6 and PI3K/Akt signaling pathways in macrophages Inhibit cell apoptosis	Suppress EMT and M2 macrophages polarization	[114]
LY294002	Rat PMCs	PMCs	Inhibit PI3K/Akt /mTOR signaling pathway and attenuating intracellular oxidative stress Decrease expression of FSP1 and α-SMA in PMCs of rat Induce polarization of M2 Promote the expression of autophagy related proteins LC3II/I, p62 and beclin-1	Inhibit fibrosis	[100]
Rapamycin					
Octreotide	Rats	——	Inhibition of TGF-β1, VEGF and MCP-1 activity	Reduce inflammation Reduce the formation of blood vessels Inhibit PF Protect peritoneal function	[118]
miR-204-5p	Rats	Macrophages	Regulation of Foxc1 Inhibit fibroblast proliferation Inhibit EMT	Inhibit fibrosis	[119]
Parthenolide	Mice/ HMrSV5	PMCs/ HMrSV5	Reduce the expression of TGF-β Inhibit phosphorylation of Smad2/3 Inhibit NF-κB/TGF-β/Smad signaling pathway	Inhibit inflammation Inhibit EMT	[122]
Potential fibrosis targets					
VPA	Mice/ RAW264.7 cells	Macrophages	Skew the phenotype of macrophages from M1 to M2 Reduce the production of inflammatory factors		[115]
Quercetin	Mice/ RAW264.7 cells	Macrophages	Inhibite the activity of NF-kB and IRF5 Clear ROS	Reduce inflammation Antioxidant	[127]
Progranulin	RAW264.7 cells	Macrophages	Inhibit inflammatory signaling pathways involved in TNF Induce polarization of M2 Reduce mRNA expression of TNF-α and iNOS Inhibit NF-κB/MAPK pathway	Inhibit inflammation	[129]
Isoliquiritin	Mice	Macrophages	Inhibit expression of genes and proteins of Mincle, and phosphorylation of Syk and NF-κB in macrophages. Decrease secretion of inflammatory factors such as IL-1β and IL-6 by macrophages	Inhibit fibrosis	[131]
Luteolin	Rats	Macrophages	Down-regulate p-STAT6 Inhibit M1 activity Induce polarization of M2 Inhibit NF-κ B, IL-17 and IL-23	Inhibit inflammation	[133]

RAW264.7: Mouse mononuclear macrophage leukemia cells; HMrSV5**:** Human peritoneal mesothelial cells; PMCs: peritoneal mesothelial cells; EMT: Epithelial-mesenchymal transition; ROS: Reactive Oxygen Species; iNOS: Inducible Nitric Oxide Synthase.

Fibrosis occurs by complex mechanisms, and the changes in peritoneal function it causes can lead directly to the inadequacy or even failure of PD. Currently, researchers are focusing on slowing down the onset of PF and prolonging the duration of PD by controlling the M1/M2 polarized macrophage ratios, inhibiting or blocking the signaling pathways involved in macrophages that are related to fibrosis, inhibiting neovascularization and introducing genes with potential therapeutic effects.

## 6. Conclusions

PF is one of the important reasons for the failure of long-term PD technology. In the process of fibrosis, pathological changes such as EMT, neovascularization and abnormal deposition of ECM appear in peritoneal tissue, which are regulated by PDGF, TGF-β, IL-1, IFN-γ, and TNF-α. TGF-β can induce CTGF synthesis through TGF-β/Smad pathway, which is an important factor in promoting fibrosis. The peritoneum contains a large number of macrophages, which is one of the main cell groups in the local defense of the abdominal cavity. Macrophages, due to their plasticity, can show 2 different functional types in response to change in the body’s microenvironment. M1 macrophages can cause histological damage in the early stage of PF and can produce pro-inflammatory factors when combined with TLR4, which maintains the peritoneum in a state of low-grade inflammation for a long time, further damage the peritoneum and reduce its filtration function [72]. INOS can increase expression of hypoxia-inducible factor-1α and MMP-9, and these factors can promote collagen remodeling and ECM deposition through different pathways [138]. M2 macrophages can inhibit inflammation and promote angiogenesis by secreting IL-1 and VEGF. As the outcome of long-term PD, PF is a complex process with multi-factorial correlation and participation of many cell types. To prolong the life of patients and delay renal failure, researchers are looking for drug targets in multiple ways to block or inhibit the process of fibrosis. TGF-β, a key factor in fibrosis, is often used as a target for intervention against fibrosis. Macrophages, which cause peritoneal inflammation and fibrosis, are regarded as a new target to delay fibrosis and are currently in the research stage. Drugs to change the microenvironment of the body, reduce inflammatory infiltration of M1 macrophages, affect the polarization of M1/M2 macrophages, induce transformation of macrophages into damage repair macrophages and reduce the occurrence and development of fibrosis are important research concerns.
